# Slow growing bacteria survive bacteriophage in isolation

**DOI:** 10.1038/s43705-023-00299-5

**Published:** 2023-09-08

**Authors:** Erin L. Attrill, Urszula Łapińska, Edze R. Westra, Sarah V. Harding, Stefano Pagliara

**Affiliations:** 1 Living Systems Institute and Biosciences, University of Exeter, Exeter, UK; 2 Environment and Sustainability Institute and Biosciences, University of Exeter, Penryn, UK; 3 Defence Science and Technology Laboratory, Porton Down, Salisbury, UK; 4 Department of Respiratory Sciences, University of Leicester, Leicester, UK

**Keywords:** Bacteriophages, Bacteria, Microbial ecology, Bacterial infection, Bacteriology

## Abstract

The interactions between bacteria and bacteriophage have important roles in the global ecosystem; in turn changes in environmental parameters affect the interactions between bacteria and phage. However, there is a lack of knowledge on whether clonal bacterial populations harbour different phenotypes that respond to phage in distinct ways and whether the abundance of such phenotypes within bacterial populations is affected by variations in environmental parameters. Here we study the impact of variations in nutrient availability, bacterial growth rate and phage abundance on the interactions between the phage T4 and individual *Escherichia coli* cells confined in spatial refuges. Surprisingly, we found that fast growing bacteria survive together with all of their clonal kin cells, whereas slow growing bacteria survive in isolation. We also discovered that the number of bacteria that survive in isolation decreases at increasing phage doses possibly due to lysis inhibition in the presence of secondary adsorptions. We further show that these changes in the phenotypic composition of the *E. coli* population have important consequences on the bacterial and phage population dynamics and should therefore be considered when investigating bacteria-phage interactions in ecological, health or food production settings in structured environments.

## Introduction

The interactions between bacteria and bacteriophage have important roles in the global ecosystem by shaping microbial populations thriving in many environments including seawater [1], soil [2] and the mammalian gut [3]. Phage are one of the major drivers of bacterial death [4], thus affecting the composition and evolution of bacterial communities in natural environments [5, 6], including the human body [7, 8]. Bacteria and phage are ubiquitous and strongly affect the environment they thrive in, therefore, deciphering bacteria-phage interactions is essential for predicting changes in natural and artificial ecosystems [5, 9, 10].

In natural environments where bacteria and phage thrive, physical and chemical parameters such as temperature, salinity, pH and nutrient availability vary in time and space. Increasing evidence suggests that such variations strongly affect bacteria-phage interactions. For example, changes in bacterial gene regulation during the colonization of the ileum and colon influences the coexistence of bacterial and phage populations [3]. Variations in temperature, salinity, pH and organic matter content affect the lysis of bacterial populations by phage in aquaculture and food production settings [11–13]. Changes in nutrient availability affect bacterial growth and increasing evidence suggests that the rate of phage population growth and burst size increase with increasing bacterial growth rate [14–16] and are life-cycle dependent [17], whereas the eclipse and latent periods reduce with increasing bacterial growth rate [14–16]. However, some phage are also capable of infecting slowly growing stationary phase or starved bacterial populations [18–21]. Moreover, lysis of the host bacterium can be delayed by the infecting phage for several hours in the event of adsorption of one or more secondary phage which leads to superinfection and lysis inhibition [22–24]. Furthermore, the spatial structure of natural environments has a profound impact on bacteria-phage interactions and the emergence of genetic resistance to phage [2, 25–32], whereas the cost of resistance can not only vary with the type of mutation [33] but also with nutrient availability [34, 35].

This wealth of understanding about the biology of bacteria and phage was obtained via measurements at the population-level that cannot dissect variations in phenotypic responses to phage from individual cells amongst a bacterial population. Whilst increasing evidence suggests that phenotypic heterogeneity within clonal bacterial populations plays a key role in many different biological processes [36–38], there is a knowledge gap in our understanding of the impact of variations in environmental parameters on the phenotypic heterogeneity in the interactions between bacteria and phage. Therefore, we set out to investigate the interaction between individual bacteria and phage in order to understand (i) whether clonal bacterial populations harbour different phenotypes that respond to phage in distinct ways, i.e. susceptible and persister bacteria [39]; (ii) whether the abundance of such phenotypes within a bacterial population is affected by variations in environmental parameters; (iii) the impact of such phenotypes on bacterial and phage population dynamics.

We used single-cell microfluidics [40–42] to perform kinetic analysis of phage infections in individual bacteria cultured as planktonic cells in spatial refuges. We chose to investigate *Escherichia coli* and its lytic phage T4 that have been extensively employed to increase our molecular understanding of the impact of the environment on bacteria-phage interactions [14–16, 43]. Moreover, we focused our study on nutrient availability, the bacterial phase of growth and phage abundance. In fact, these environmental parameters often vary over time in natural structured environments, can be easily manipulated in applied settings and are closely linked with each other. Understanding how variations in nutrient availability, bacterial phase of growth and phage abundance shape phenotypic heterogeneity in bacteria-phage interactions is of major importance from an ecological [4, 5, 9, 44] and applied standpoint [45–48].

## Methods

### Materials

All materials were purchased from Fisher Scientific or Sigma-Aldrich unless otherwise stated. Lysogeny broth (LB) was purchased from Melford and consisted of 10 g/L tryptone, 5 g/L yeast extract and 10 g/L NaCl. *E. coli* strain BW25113 was purchased from Dharmacon (GE Healthcare). M9 minimal medium was prepared to the following recipe (7 g l^−1^ Na_2_HPO_4_, 3 g l^−1^ KH_2_PO_4_, 1 g l^−1^ NH_4_Cl, 0.5 g l^−1^ NaCl, 1 mM thiamine hydrochloride). T4 phage ATCC-11303-B4 was purchased from LGC Standards and propagated and stored as previously described [31].

### Bacterial culture and phage propagation

Overnight cultures were prepared as previously described [31]. Briefly, single colonies of *E. coli* BW25113 were picked from LB agar and grown in 200 mL fresh LB shaking at 200 rpm and 37 °C overnight (i.e. 17 h). To propagate T4 phage, the bacterial overnight cultures were diluted 1:1000 in LB and grown for 4 h until they were in mid-exponential phase at approximately 10^6^ cells ml^−1^. T4 was then added at an MOI of 1 and left to incubate overnight at 37 °C. 10% v-v chloroform was added and lightly agitated for 15 min. The samples were then centrifuged at 4000 rpm for 30 min and the supernatant filtered twice through a 0.22 µm filter and stored at 4 °C.

### Infection assays in the mother machine

The mother machine microfluidic device was engineered, assembled and used to conduct single cell time-lapse microscopy experiments as previously reported [31, 42, 49]. Briefly, overnight *E. coli* cultures were concentrated to an OD_600_ of 50 through centrifugation and resuspension of the pellet in spent medium that was obtained by filtering the supernatant from the overnight culture twice. A 2 µl aliquot of the concentrated bacterial suspension above was loaded into the main delivery channel of the mother machine device, and the chip incubated at 37 °C for 20 min to ensure approximately 50% of the mother machine spatial refuges were loaded with 1–2 bacteria. The microfluidic chip was connected to fluorinated ethylene propylene tubing and mounted onto an inverted microscope within an environmental chamber held at 37 °C as previously described [50]. In order to study bacteria in the lag phase, bacteria were treated with T4 phage diluted to a concentration of either 10^7^ or 10^9^ plaque forming units (PFU) ml^−1^ in either 10:90 LB:M9 (V:V) or in LB only immediately after addition to the mother machine. To target exponentially growing bacteria, bacteria were first grown in LB for 3 h and then exposed to T4 phage at a concentration of 10^7^ PFU ml^−1^ in 10:90 LB:M9. To target bacteria in stationary phase, bacteria were treated with T4 phage at a concentration of 10^7^ PFU ml^−1^ in spent medium obtained by filtering the supernatant of the same overnight *E. coli* culture twice immediately after addition to the mother machine. In all cases, T4 phage was actively supplied into the mother machine for 2 h, unless specified otherwise, at a flow rate of 100 µl h^−1^. After 2 h, fresh LB medium only was flowed through the device at 100 µl h^−1^ for 22 h. During the 2 h of active phage supply in the mother machine, bacteria experienced both active and passive exposure to phage, the latter originating from the lysis of bacteria that had previously been infected by phage. During the following 22 h exposure to LB only, bacteria experienced exclusively passive phage exposure. Brightfield images were collected at hourly time-points using an exposure time of 0.03 s. At 24 h propidium iodide (PI) in LB (1:1000, V:V) was introduced into the mother machine device and images were taken using a TRITC filter and a green LED at 100% intensity. Each experiment was performed in biological triplicate.

In order to measure the phage population dynamics, the mother machine outflow was collected hourly into tubes containing 1 mg/ml kanamycin to prevent phage replication and serially diluted in LB and spotted on plates prepared as follows: 4 ml of liquid 0.5% LB agar was pipetted onto plates containing 1.5% LB agar. 200 μL of an overnight *E. coli* culture was added to each plate, followed by 100 μL of each phage dilution. Plates were incubated overnight at 37 °C and plaques were enumerated. Only plates containing 30–300 plaques were counted.

In order to measure the number of particles reaching individual spatial refuges, we used fluorescent nanoparticles of a similar size to the T4 bacteriophage. These particles were amine-modified polystyrene beads conjugated to orange fluorescent dye with diameter range 100–20 nm [51] and were purchased from Sigma-Aldrich. These nanoparticles were injected into the mother machine at a concentration of either 10^7^ or 10^9^ particles ml^−1^ at a flow rate of 100 µl h^−1^ and their diffusion in 20 spatial refuges was continuously imaged for 2 h via a Texas red filter, a green LED at 100% intensity, and a camera exposure time of 0.1 s. This experiment was performed in triplicate.

### Assessment of bacterial genetic resistance to T4

During the above-described infection assays in the mother machine, the outflow from the chip was collected at hourly time points. Each outflow sample had a volume of around 100 µl since we used a flow rate of 100 µl per hour in our experiments. Each outflow sample contained either bacteria and growth medium in control experiments, or bacteria, phage and growth medium in infection experiments. In order to test whether bacteria exposed to phage within the mother machine had become resistant to phage, sterile loops were dipped into each outflow sample from control and infection experiments and streaked across plates containing T4 at a concentration of approximately 10^9^ PFU ml^−1^. We did not observe formation of bacterial colonies in any of the plates, suggesting that genetic resistance to T4 does not emerge within *E. coli* exposed to T4 within the mother machine and within our 24 h experimental time frame.

### Image and data analysis

For each environmental condition, we measured the fate of individual bacteria in each of 200 separate refuges of the mother machine device from biological triplicate at hourly time points. Specifically, quantitative information on the bacterial population dynamics was extracted by loading the time lapse microscopy images of the bacteria-hosting channels into ImageJ. The number of cells present in each channel was counted at each time point, and the cell fate, live, lysed or PI stained, was assessed using bright field images and PI staining images at t = 24 h. Since each mother machine channel accommodates up to eight bacteria, around 5% of the bacterial progeny was pushed out from the open end of the hosting channels during the single-cell assay and the fate of such cells during T4 exposure could not be directly measured. Therefore, predictions of the probabilities of the possible fate of these cells were made based on the measurable fate for the remaining 95% of the population.

The survival fraction in each hosting refuge at each time point was determined as the bacterial counts in the hosting refuge at any given time point divided by the count at t = 0 in the same refuge. The lysed fraction was determined as the number of bacteria that lysed in each refuge at each time point divided by the number of bacteria present in the same refuge at that time point. Means and standard errors for both quantities were then calculated by averaging measurements performed over 200 different hosting channels from three independent biological replicates. The overall surviving fraction in each experiment was determined as the total number of bacteria that survived by 24 h divided by the total number of bacteria counted during the experiment. The overall susceptible fraction in each experiment was determined as the total number of bacteria that lysed or stained with PI divided by the total number of bacteria counted during the experiment. Means and standard errors for both quantities were obtained by averaging data from biological triplicates.

The overall phenotype of each channel was determined and classified into one of 8 distinct phenotypes: bacteria that doubled and survived with their entire progeny in the hosting spatial refuge and that we called survivors without lysis; bacteria that doubled and survived with some of their progeny but with evidence of lysis in the hosting spatial refuge and that we called survivors with lysis; bacteria that did not double and survived and that we called survivors without doubling; bacteria that doubled with only the daughter cell at the dead-end of the hosting spatial refuge surviving and that we called old pole survivors since they consistently inherited the old pole of the progenitor cell [52]; bacteria that doubled with only one of the daughter cells that were not at the dead-end of the hosting spatial refuge surviving and that we called new pole survivors since they did not consistently inherit the old pole of the progenitor cell [52]; bacteria that lysed without doubling; bacteria that doubled and lysed within 7 h; bacteria that doubled and lysed within 24 h. The fraction of each phenotype was calculated for each hosting channel and the means and standard errors were obtained by averaging data from 200 channels from biological triplicate.

The number of generations each susceptible cell underwent before lysis was also calculated. Data were tested for normality using a Shapiro Wilks test and differences between the number of generations before death compared through Mann-Whitney tests. Linear regressions were conducted on the survival fractions from t = 2 h to t = 7 h post phage addition, to extrapolate the intercept to the *x*-axis. The slopes and intercepts were then compared.

Quantitative information on the diffusion of fluorescent nanoparticles in the structured environment was extracted by loading the time lapse fluorescence microscopy images in ImageJ and counting the number of particles reaching each spatial refuge during each 2 h long exposure. In order to exclude spurious noise [53, 54], we retained the events for which a particle explored a spatial refuge for three consecutive frames (i.e. >0.3 s). For each of the analysed spatial refuges, we evaluated the relative frequency for which the spatial refuge was empty, or contained one or more particles at any given time; for each relative frequency we calculated the mean and standard error of the measurements obtained in 20 spatial refuges from three independent experiments.

## Results

### Bimodal phenotypic survival of *E. coli* to T4 exposure

**Fig. 1:**
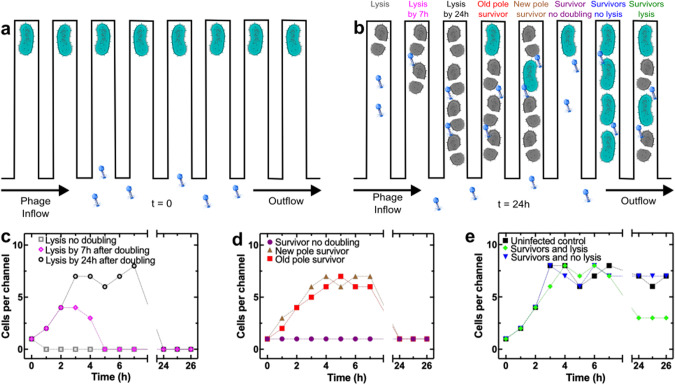
Phenotypic heterogeneity in the interaction between *E. coli* and the T4 phage. Schematic illustrating the experimental design: (**a**) individual bacteria confined in microfluidic spatial refuges with phage being delivered to the microfluidic environment at t = 0 and (**b**) eight different phenotypic responses to phage recorded within 24 h, from left to right: lysis without doubling, lysis by 7 h or 24 h after doubling, individual old pole survivor after doubling, individual new pole survivor after doubling, individual survivor without doubling, multiple survivors without lysis, multiple survivors with evidence of lysis. **c**–**e** Corresponding representative temporal dependence of bacterial density per refuge for each of the phenotypes above. Dotted lines are guides for the eye. These measurements are representative of experiments carried out in 200 refuges of the structured mother machine environment in biological triplicate. Corresponding representative time-lapse microscopy images are presented in Fig. S1. Quantitative information extracted from the full image sets are presented in Figs. 2–4.

In order to study the interactions between *E. coli* and T4, we used our recently introduced high-throughput, microfluidics-based platform to perform kinetic analysis of antibiotic efficacy [37, 41, 42, 55, 56], molecular accumulation [36, 38, 40, 57, 58] and phage infections in individual bacterial cells [31, 59]. Briefly, we used a microfluidic mother machine device [60] equipped with thousands of compartments physically separated from each other that represent spatial refuges where bacteria can grow, each refuge initially hosting one bacterium (Fig. 1a). When phage were actively supplied via microfluidics to the environment around the refuges, we discovered eight different phenotypic bacterial responses that broadly fall within three categories: bacterial lysis, bacterial survival in isolation, collective bacterial survival (Fig. 1b). Specifically, in some refuges individual bacteria were lysed by T4 without doubling or all progeny was lysed by T4 after doubling either by t = 7 h or t = 24 h following the addition of the phage (Fig. 1b–c, Fig. S1a–c). In other refuges bacteria first doubled and then survived phage in isolation: some surviving bacteria were positioned at the dead-end of the refuge and thus consistently inherited the original old pole of the progenitor cell during doubling [52], whereas other bacteria were not positioned at the dead-end of the compartment and thus did not consistently inherit the old pole of the progenitor cell during doubling (Figs. 1b, 1d, S1d–e). In other refuges bacteria were not lysed during phage treatment and did not double but survived in isolation in their compartment (Figs. 1b, 1d, S1f). In other refuges we found collective survival to phage, where *E. coli* doubled and either none of the progeny was lysed by phage or only some of the progeny was lysed by phage (Figs. 1b, 1e, S1h–i). These surviving bacteria were not genetically resistant to T4 as none of the bacteria collected in the outflow of the mother machine grew on LB agar plates containing T4.

Taken together, these data demonstrate that *E. coli* can survive exposure to T4 without acquiring genetic mutations via two main distinct phenotypes: collective survival or isolated survival in spatial refuges. In the following we set out to investigate whether the relative abundance of these distinct phenotypes is stochastic or can be controlled by manipulating the environment around the bacteria.

### The lag phase of growth favours isolated survival to phage

**Fig. 2:**
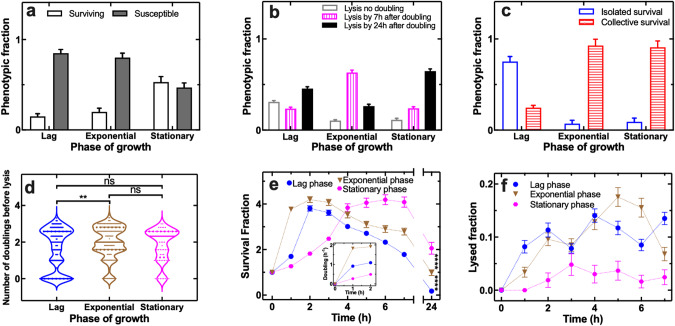
The lag phase of growth favours isolated survival to phage and better bacterial clearance. **a**–**d** Quantitative comparisons of the efficacy of a 2 h treatment with 10^7^ T4 phage ml^-1^ against *E. coli* in the lag, exponential or stationary phase of growth in terms of the (**a**) overall fraction of susceptible (filled bars) and surviving bacteria (empty bars), (**b**) fraction of bacteria that lysed without doubling (empty bars), lysed by 7 h after doubling (vertically patterned bars), or lysed by 24 h after doubling (filled bars), (**c**) fraction of isolated (empty bars) vs collective survival (horizontally patterned bars), (**d**) number of doublings before lysis. **e**–**f** Temporal dependence of (**e**) the survival and (**f**) the lysed fraction for bacteria in the lag (blue circles), exponential (brown triangles) or stationary phase of growth (magenta hexagons) hosted in the mother machine compartments and treated with 10^7^ T4 phage ml^−1^ for 2 h. Lag and exponential *E. coli* were treated for 2 h with phage suspended in a 9:1 M9:LB mixture, whereas stationary phase bacteria were treated for 2 h with phage suspended in spent medium harvested from an overnight *E. coli* culture. In all three cases, LB medium only was delivered in the mother machine from t = 2 h onwards instead of medium containing T4 phage. Inset in (**e**): mean doubling rate of lag (blue circles), exponential (brown triangles) or stationary phase *E. coli* (magenta hexagons) during the 2 h treatment with 10^7^ T4 phage ml^−1^. Measurements were carried out in 200 channels of the structured mother machine environment in biological triplicate, means and standard errors of the mean were calculated and used in panels (**e**) and (**f**). Very small error bars cannot be visualised due to overlap with the datapoints. Dotted lines are guides for the eye.

Firstly, we set out to determine the impact of the phase of bacterial growth on the relative abundance of the eight phenotypic responses to phage introduced above. We used a 2 h active treatment with 10^7^ phage ml^−1^ against bacteria in the lag, exponential or stationary phases of growth and altered the microfluidic supply to growth medium only at t = 2 h for a further 22 h with phage persisting in the mother machine originating exclusively from previously infected cells. We found that the fraction of bacteria surviving phage was significantly higher for bacteria in the stationary phase of growth compared to the lag or exponential phases (Fig. 2a) and that bacterial lysis by phage was slower in the lag or stationary phases compared to the exponential phase (Fig. 2b) with an increase in the number of doublings before lysis when phage was added to bacteria in the exponential phase of growth (Fig. 2d). Remarkably, we discovered a switch from isolated survival for lag phase bacteria to collective survival for exponential and stationary phase bacteria (Fig. 2c). For lag phase bacteria the most common surviving phenotype was the old pole survivor, whereas bacteria doubling and surviving without evidence of lysis was the most common phenotype for both exponential and stationary phase bacteria (Fig. S2a).

The bacterial survival fraction and doubling rate initially increased to a greater extent when treatment was carried out against exponential phase bacteria compared to lag or stationary phase bacteria (Fig. 2e); as a consequence, the phage population expanded to a greater extent in exponentially growing bacteria compared to lag or stationary phase bacteria (Fig. S3a). After two hours, the bacterial population started to decrease with a longer predicted time of population extinction for exponential compared to lag phase bacteria due to the prevalence of collective phenotypic survival within exponential phase bacteria (Fig. 2e). Accordingly, the phage population reduced earlier and more steeply in exponential compared to lag phase bacteria (Fig. S2a). The survival fraction continued to increase, whilst the lysed fraction and phage counts remained low in the case of stationary phase bacteria (Fig. 2e, f and Fig. S2a) due to collective phenotypic survival. Therefore, at t = 24 h the bacterial survival fraction was highest for stationary or exponential phase bacteria, predominantly displaying collective survival, compared to lag phase bacteria, predominantly displaying isolated survival (Fig. 2e).

Our data describing the dynamics of phage populations explain the observed dependence of survival to phage on the bacterial phase of growth. Bacteria that had been supplied with phage whilst in stationary phase had higher chances of collective phenotypic survival compared to lag phase bacteria because of significantly lower phage levels available for infection (Fig. S2a). Bacteria that had been supplied with phage whilst in exponential phase were instead surrounded by a larger number of phage compared with lag phase bacteria (Fig. S2a). Therefore, exponentially growing bacteria already infected by a phage had a greater probability to be infected by a second phage within few minutes from the first infection, possibly triggering lysis inhibition. In line with this hypothesis, only a minority of bacteria were lysed by phage whilst the phage population was at its maximal levels (Fig. 2b).

### Increasing nutrient availability favours collective survival to phage

**Fig. 3:**
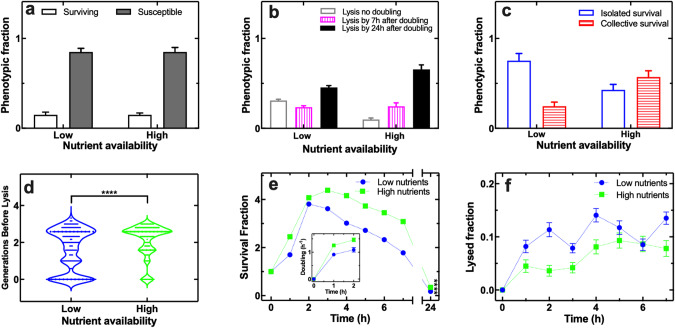
Increased nutrient availability triggers a switch to phenotypic collective survival and reduces bacterial clearance by phage. **a**–**d** Quantitative comparisons of a 2 h treatment with 10^7^ phage ml^−1^ at low (i.e. 9 : 1 minimal medium M9 : LB mixture) or high nutrient availability (i.e. LB) in terms of (**a**) overall fraction of susceptible (filled bars) and surviving bacteria (empty bars), (**b**) fraction of bacteria that lysed without doubling (empty bars), lysed by 7 h after doubling (vertically patterned bars), or lysed by 24 h after doubling (filled bars), (**c**) fraction of isolated survival (empty bars) vs collective survival (horizontally patterned bars), (**d**) number of doublings before lysis. LB medium only was delivered in the mother machine from t = 2 h onwards. **e**–**f** Temporal dependence of (**e**) the survival fraction and (**f**) the lysed fraction for *E. coli* hosted in the mother machine compartments and treated with 10^7^ phage ml^−1^ at low (blue circles) or high nutrient availability (green squares). Inset in (e): mean doubling rate during a 2 h treatment with 10^7^ phage ml^−1^ at low (blue circles) or high nutrient availability (green squares). Measurements were carried out in 200 channels of the structured mother machine environment in biological triplicate, means and standard errors of the mean were calculated and used in panels (**e**) and (**f**). Very small error bars cannot be visualised due to overlap with the datapoints. Dotted lines are guides for the eye.

Next, we set out to determine the impact of nutrient availability on the relative abundance of the above introduced eight phenotypic responses to phage. The phage population initially amplified to a greater extent in LB compared to a nutrient poorer mixture of LB and minimal medium at a ratio 1:9 (Fig. S3b). Accordingly, we observed a delay in bacterial lysis (Fig. 3b), a switch from isolated to collective survival (Fig. 3c) and an increase in the number of doublings before lysis (Fig. 3d) at high nutrient compared to low nutrient availability that also allowed for significantly faster bacterial growth in the absence of phage (Fig. S4). The survival fraction and doubling rate was initially higher in the presence of high nutrient compared to low nutrient availability (Fig. 3e), whereas the lysed fraction was higher at low nutrient availability (Fig. 3f). Moreover, the phage population reduced earlier and more steeply at high compared to low nutrient availability from t = 3 h onwards (Fig. S3B). As a result, increased nutrient availability also reduced the predicted time of extinction of the bacterial population (Fig. 3e) with a significantly higher surviving population fraction at t = 24 h (Fig. 3e).

Taken together, these data further support our hypothesis that collective phenotypic survival to phage emerges during periods in which phage originating from bacterial lysis are more abundant because of increased nutrient availability and bacterial growth rate.

### Increasing phage abundance favours collective bacterial survival to phage

**Fig. 4:**
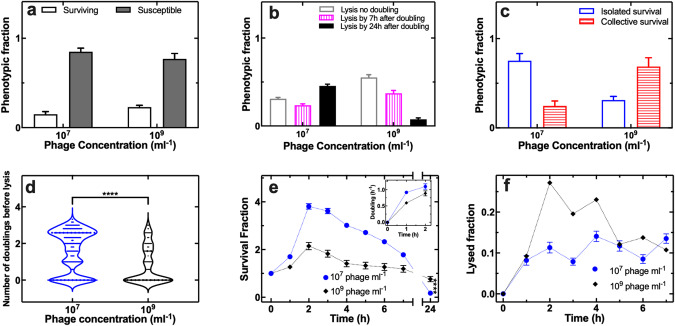
Increasing phage concentration favours collective bacterial survival to phage and reduces bacterial clearance by phage. **a**–**d** Quantitative comparisons of the efficacy of 10^7^ or 10^9^ T4 phage ml^−1^ treatment in terms of (**a**) overall fraction of susceptible (filled bars) and surviving bacteria (empty bars), (**b**) fraction of bacteria that lysed without doubling (empty bars), lysed by 7 h after doubling (vertically patterned bars), or lysed by 24 h after doubling (filled bars), (**c**) fraction of isolated survival (empty bars) vs collective survival (horizontally patterned bars), (**d**) number of doublings before lysis. **e**, **f** Temporal dependence of (**e**) the survival fraction and (**f**) the lysed fraction for *E. coli* hosted in the mother machine compartments and treated with 10^7^ (blue circles) or 10^9^ T4 phage ml^−1^ for 2 h (black diamonds). LB medium only was delivered in the mother machine from t = 2 h onwards. Inset in (**e**): mean bacterial doubling rate during the 2 h treatment with 10^7^ (blue circles) or 10^9^ T4 phage ml^−1^ (black diamonds). Measurements were carried out in 200 channels of the structured mother machine environment in biological triplicate, means and standard errors of the mean were calculated and used in panels (**e**) and (**f**). Very small error bars cannot be visualised due to overlap with the datapoints. Dotted lines are guides for the eye.

Next, we set out to determine the impact of phage abundance on the relative frequency of the above introduced eight phenotypic responses to phage. We found that the fractions of surviving bacteria for treatment with 10^7^ and 10^9^ phage ml^−1^ were not significantly different (Fig. 4a). However, increasing the phage concentration accelerated bacterial lysis (Fig. 4b). In accordance with our lysis inhibition hypothesis, at the low dose regime isolated survival was predominant; in contrast, collective survival was predominant at the high dose regime (Fig. 4c). Moreover, we found a lower initial bacterial population expansion (Fig. 4e), a slower bacterial doubling rate (Fig. 4e) and a reduction in the number of doublings before lysis (Fig. 4d,) during the high dose regime compared to the low dose regime. These data suggest that a high phage dose regime is more efficient in limiting the initial bacterial growth measured at the low phage regime. However, we then observed a less steep decrease in survival fraction at the high dose regime which led to a higher final survival fraction at the high dose regime at t = 24 h (Fig. 4e). This higher survival was due to a 5-fold reduction in the phage population (from t = 0 to t = 3 h, Fig. S1c) when the phage input was high, which led to the prevalence of collective bacterial survival under the high phase dose regime. In contrast, the phage population reached a maximum amplification of a factor of 10 compared to the input population in the case of low phage input (Fig. S1c).

We observed similar phenotypic responses to phage when active phage treatment was extended from a 2 h to a 24 h period (Fig. S5), suggesting that the newly discovered phenotypic switch from isolated to collective survival to phage is not dictated by the length of time in which phage are actively supplied to the bacterial environment. Furthermore, using fluorescent nanoparticles of dimensions similar to bacteriophage T4, we found that when these nanoparticles were injected in the mother machine device at a concentration of 10^7 ^ml^−1^, the probability that a compartment was simultaneously explored by two or more particles was low (i.e. 0.25, Fig. S4a); in contrast, when these nanoparticles were injected in the mother machine device at a concentration of 10^9 ^ml^−1^, the probability that a compartment was simultaneously explored by two or more particles was high (i.e. 0.7, Fig. S6b).

## Discussion

Variations in physical and chemical parameters affect bacteria–phage interactions [3, 11–17] which in turn shape the ecology and evolution of microbial communities [1–3]. A diverse arsenal of genetic mechanisms permitting bacteria to resist phage across different environmental conditions have been reported [2, 5, 25–28]. In contrast the impact of variations in environmental parameters on the phenotypic heterogeneity in the interactions between bacteria and bacteriophage remains to be investigated. In fact, investigations of bacteria–phage interactions [5, 61, 62] and the evolution of resistance to phage [63–67] are often only carried out at the genetic level on bacteria growing in one defined environmental condition and often do not consider how these interactions may in turn depend on the environment. Here we present novel understanding of the different phenotypic responses to phage harboured within clonal bacterial populations and on the impact of variations in nutrient availability, the bacterial phase of growth and phage abundance on the frequency of these bacterial phenotypes.

Remarkably, we discovered that an increase in either nutrient availability, or bacterial growth rate, or phage abundance drives a drastic phenotypic switch from isolated survival to collective survival in spatial refuges (Fig. S7), demonstrating that these three interlinked environmental factors have a profound impact not only on bacterial populations [14–16] but also on the phenotypic composition within a clonal population at least in the bacterium *E. coli* and phage T4 pair. Because of such switch to collective phenotypic survival, we found that bacterial populations growing at high nutrient availability or in the exponential phase of growth displayed higher survival to phage T4 compared to bacterial populations thriving at low nutrient availability or in the lag phase of growth. In contrast, in well-mixed environments, survival of bacterial populations to phage decreases with nutrient availability and bacterial growth rate [14, 16, 17], a discrepancy on which we expand below.

The phenotypic switch between isolated and collective survival is possibly controlled and mediated by phage via lysis inhibition since we consistently observed this switch when phage was actively provided at a high concentration, or a high concentration of phage originated from infected bacteria as was the case for bacteria infected in the exponential phase of growth or in the lag phase of growth but with nutrient supplementation (Fig. S7). Moreover, our experiments using nanoparticles suggested that the probability of super-infection increases with phage abundance. Super-infection is known to cause lysis inhibition for phage T4 [24, 68], where lysis of a super-infected *E. coli* cell can be delayed for several hours. During this period the cell may grow and double [23], as we observed in our study with the collective survival phenotype. Therefore, it is conceivable that the above reported discrepancy in lysis of bacterial populations by phage between well-mixed and structured environments is due to enhanced lysis inhibition in the latter environment, because of the physical confinement and proximity of phage and bacteria. However, it is also conceivable that in the presence of a higher number of fast growing bacteria in spatial refuges, phage slow down infection; accordingly, T4 phage adsorption constant decreases with bacterial growth rate in chemostats [15].

Alternatively, the collective survival phenotype could be attributed to persistence to phage where the expression of the phage lytic genes is suppressed in non-growing persistent bacteria and the infecting phage resumes the process of gene expression and causes cell lysis when bacteria switch back to normal growth [39]. However, in contrast to this previous report we found that the collective survival phenotype grew also in the presence of phage, suggesting that our newly discovered collective survival phenotype is underpinned by a distinct mechanism with respect to persistence to phage; the observed phenotype survivor without doubling could be instead attributed to persistence to phage.

It is also conceivable that bacteria that had been actively supplied with phage at high nutrient availability or in the exponential phase of growth were in a more active physiological state and, therefore, created more progeny and initiated phenotypic defences to counteract phage attacks, for example, via a rapid downregulation of the phage receptors [31]. However, this scenario does not explain why we observed a switch from isolated to collective survival at increased phage abundance while maintaining the same nutrient availability and phase of growth.

This newly discovered phage-controlled switch reminds us of the reversible transition between lysis and lysogeny by temperate phage. This transition often depends on the density of susceptible hosts in the environment and is based on phage communication [69, 70] and monitoring of bacterial quorum-sensing systems [71–73]. In fact, experimental and theoretical studies using temperate phage (e.g. *E. coli* phage λ, *B. subtilis* phage phi3T) in well-mixed environments have demonstrated that when the multiplicity of infection is low and the number of susceptible hosts is high, lysis followed by horizontal transmission is the preferred strategy for temperate phage; conversely, when the multiplicity of infection is high and the number of susceptible hosts is low, the phage benefits more from investing into lysogeny and vertical transmission [69, 74–76].

Similarly, using the lytic phage T4 and a structured environment, we found that when the multiplicity of infection is low, phage lyse infected cells and bacteria predominantly survive in isolation in spatial refuges; conversely, when the multiplicity of infection is high phage do not lyse infected cells and bacteria predominantly survive together with their clonal kin in spatial refuges. Noteworthy, super-infection exclusion, whereby phage infected cells became immune to subsequent infection, has been recently reported as a defence strategy in environmental bacteria [77]. Therefore, if confirmed for other bacterial species dwelling in structured soil or aquatic environments, this phenotypic switch might have important consequences on the ecology of microbial communities. For example, in nutrient depleted spatial refuges isolated survivors of a bacterial species infected by its phage will be quickly outcompeted by other bacterial species that are not affected by phage. However, seasonal variations in nutrient availability might change this scenario allowing this bacterium to persist in the spatial refuge due to collective phenotypic survival to phage.

We also discovered that the predominant phenotype among bacteria surviving in isolation in spatial refuges was the old pole survivor, that is the cell that was positioned at the dead-end of the refuge and thus consistently inherited the original old pole of the progenitor cell during doubling. According to previous studies these cells grow more slowly compared with new pole cells, possibly due to asymmetric partitioning of damaged or beneficial components from the mother to its daughter cells [52, 78]. It is conceivable that slower growth protects old pole bacteria from phage as in the case of persistence to phage [39] or leads to a lower expression of the outer membrane porin OmpC [79] which is one of the phage receptors. However, it is also conceivable that at low phage abundance, new pole cells are infected by the incoming phage first and effectively screen old pole cells that are not reached by the phage. In support of this hypothesis, only a minority of old pole cells survived when phage abundance was increased.

It is also worth noting that in well-mixed environments phage abundance and multiplicity of infection change over time, phage virulence and horizontal transmission being highest at the onset of an epidemic and reducing as the epidemic depletes the pool of susceptible hosts [74]. Accordingly, in the structured environment we found that the T4 population initially expands when the susceptible hosts are plentiful but subsequently declines when the susceptible hosts become scarce. Moreover, such decline starts earlier, and its slope is steeper when the multiplicity of infection is high. Importantly, in the structured environment the multiplicity of infection also varies in space and accordingly we found evidence of both collective and isolated survival to phage under the same environmental conditions, although low multiplicity of infection significantly favoured isolated bacterial survival, whereas high multiplicity of infections significantly favoured collective bacterial survival.

Our new findings highlight the importance of investigating the impact of environmental variations, such as the structure of the environment and nutrient availability, on bacteria-phage interactions. In fact, in naturally structured environments, the level of genetic resistance evolution is lower compared to within well-mixed environments [2, 25, 28, 30, 80]. However, our data demonstrate that, in structured environments, bacteria phenotypically survive phage exposure without evolving genetic resistance and that nutrient availability drives a phenotypic switch from isolated to collective survival which is possibly controlled by phage via lysis inhibition. Although our experiments are a necessary simplification of a more complex reality, our novel findings advance our current understanding of how variations in environmental parameters dictate bacteria-phage interactions, and should be taken into account in ecological, health or food production settings in structured environments [26–28, 30, 80, 81].

### Supplementary information

Figure S1

Figure S2

Figure S3

Figure S4

Figure S5

Figure S6

Figure S7

## Data Availability

All data generated or analysed during this study are included in this published article and its supplementary information files.

## References

[1] Aylward FO, Boeuf D, Mende DR, Wood-Charlson EM, Vislova A, Eppley JM (2017). Diel cycling and long-term persistence of viruses in the ocean’s euphotic zone. Proc Natl Acad Sci USA.

[2] Gómez P, Buckling A (2011). Bacteria-phage antagonistic coevolution in soil. Science (80-).

[3] Lourenço M, Chaffringeon L, Lamy-Besnier Q, Titécat M, Pédron T, Sismeiro O (2022). The gut environment regulates bacterial gene expression which modulates susceptibility to bacteriophage infection. Cell Host Microbe.

[4] Suttle CA (2007). Marine viruses - major players in the global ecosystem. Nat Rev Microbiol.

[5] Chevallereau A, Pons BJ, van Houte S, Westra ER (2022). Interactions between bacterial and phage communities in natural environments. Nat Rev Microbiol.

[6] Hansen MF, Svenningsen SL, Røder HL, Middelboe M, Burmølle M (2019). Big impact of the tiny: bacteriophage–bacteria interactions in biofilms. Trends Microbiol.

[7] Gregory AC, Zablocki O, Zayed AA, Howell A, Bolduc B, Sullivan MB (2020). The gut virome database reveals age-dependent patterns of virome diversity in the human gut. Cell Host Microbe.

[8] Mirzaei MK, Maurice CF (2017). Ménage à trois in the human gut: interactions between host, bacteria and phages. Nat Rev Microbiol.

[9] Clokie MRJ, Millard AD, Letarov AV, Heaphy S (2011). Phages in nature. Bacteriophage.

[10] Weinbauer MG (2004). Ecology of prokaryotic viruses. FEMS Microbiol Rev.

[11] Silva YJ, Costa L, Pereira C, Cunha Â, Calado R, Gomes NCM (2014). Influence of environmental variables in the efficiency of phage therapy in aquaculture. Microb Biotechnol.

[12] Fister S, Robben C, Witte AK, Schoder D, Wagner M, Rossmanith P (2016). Influence of environmental factors on phage-bacteria interaction and on the efficacy and infectivity of phage P100. Front Microbiol.

[13] Tokman JI, Kent DJ, Wiedmann M, Denes T (2016). Temperature significantly affects the plaquing and adsorption efficiencies of Listeria phages. Front Microbiol.

[14] Hadas H, Einav M, Zaritsky A (1994). Bacteriophage T4 development depends on the physiology of its host E. coli. Microbiology.

[15] Nabergoj D, Modic P, Podgornik A (2018). Effect of bacterial growth rate on bacteriophage population growth rate. Microbiologyopen.

[16] Šivec K, Podgornik A (2020). Determination of bacteriophage growth parameters under cultivating conditions. Appl Microbiol Biotechnol.

[17] Storms ZJ, Brown T, Cooper DG, Sauvageau D, Leask RL (2014). Impact of the cell life-cycle on bacteriophage T4 infection. FEMS Microbiol Lett.

[18] Bryan D, El-Shibiny A, Hobbs Z, Porter J, Kutter EM (2016). Bacteriophage T4 infection of stationary phase E. coli: life after log from a phage perspective. Front Microbiol.

[19] Golec P, Karczewska-Golec J, Loś M, Wegrzyn G (2014). Bacteriophage T4 can produce progeny virions in extremely slowly growing Escherichia coli host: comparison of a mathematical model with the experimental data. FEMS Microbiol Lett.

[20] Melo LDR, França A, Brandão A, Sillankorva S, Cerca N, Azeredo J (2018). Assessment of Sep1virus interaction with stationary cultures by transcriptional and flow cytometry studies. FEMS Microbiol Ecol.

[21] Briggiler Marcó M, Reinheimer J, Quiberoni A (2015). Phage adsorption and lytic propagation in Lactobacillus plantarum: could host cell starvation affect them?. BMC Microbiol.

[22] Abedon S (2019). Look who’s talking: T-even phage lysis inhibition, the granddaddy of virus-virus intercellular communication research. Viruses.

[23] Doermann AH (1947). Lysis inhibition with Escherichia coli bacteriophages. J Bacteriol.

[24] Dressman HK, Drake JW (1999). Lysis and lysis inhibition in bacteriophage T4: rV mutations reside in the holin t gene. J Bacteriol.

[25] Hernandez CA, Koskella B (2019). Phage resistance evolution in vitro is not reflective of in vivo outcome in a plant‐bacteria‐phage system*. Evolution (NY).

[26] Abedon ST, Yin J. 2008. Impact of spatial structure on phage population growth, p. 94–113. In Bacteriophage Ecology: Population Growth, Evolution, and Impact of Bacterial Viruses (Advances in Molecular and Cellular Microbiology). Cambridge University Press.

[27] Brockhurst MA, Buckling A, Rainey PB (2006). Spatial heterogeneity and the stability of host-parasite coexistence. J Evol Biol.

[28] Eriksen RS, Svenningsen SL, Sneppen K, Mitarai N (2017). A growing microcolony can survive and support persistent propagation of virulent phages. Proc Natl Acad Sci USA.

[29] Heilmann S, Sneppen K, Krishna S (2012). Coexistence of phage and bacteria on the boundary of self-organized refuges. Proc Natl Acad Sci USA.

[30] Vidakovic L, Singh PK, Hartmann R, Nadell CD, Drescher K (2017). Dynamic biofilm architecture confers individual and collective mechanisms of viral protection. Nat Microbiol.

[31] Attrill EL, Claydon R, Łapińska U, Recker M, Meaden S, Brown AT (2021). Individual bacteria in structured environments rely on phenotypic resistance to phage. PLOS Biol.

[32] Brockhurst MA, Rainey PB, Buckling A (2004). The effect of spatial heterogeneity and parasites on the evolution of host diversity. Proc R Soc B Biol Sci.

[33] Buckling A, Wei Y, Massey RC, Brockhurst MA, Hochberg ME (2006). Antagonistic coevolution with parasites increases the cost of host deleterious mutations. Proc R Soc B Biol Sci.

[34] Dietrich R, Ploss K, Heil M (2005). Growth responses and fitness costs after induction of pathogen resistance depend on environmental conditions. Plant Cell Environ.

[35] Lopez-Pascua LD, Buckling A (2008). Increasing productivity accelerates host–parasite coevolution. J Evol Biol.

[36] Glover G, Voliotis M, Łapińska U, Invergo BM, Soanes D, O’Neill P (2022). Nutrient and salt depletion synergistically boosts glucose metabolism in individual Escherichia coli cells. Commun. Biol.

[37] Goode O, Smith A, Zarkan A, Cama J, Invergo BM, Belgami D (2021). Persister Escherichia coli cells have a lower intracellular pH than susceptible cells but maintain their pH in response to antibiotic treatment. MBio.

[38] Łapińska U, Voliotis M, Lee KK, Campey A, Stone MRL, Phetsang W (2022). Fast bacterial growth reduces antibiotic accumulation and efficacy. Elife.

[39] Pearl S, Gabay C, Kishony R, Oppenheim A, Balaban NQ (2008). Nongenetic individuality in the host–phage interaction. PLoS Biol.

[40] Cama J, Voliotis M, Metz J, Smith A, Iannucci J, Keyser UF (2020). Single-cell microfluidics facilitates the rapid quantification of antibiotic accumulation in Gram-negative bacteria. Lab Chip.

[41] Goode O, Smith A, Łapińska U, Attrill E, Carr A, Metz J (2021). Heterologous protein expression favors the formation of protein aggregates in persister and viable but nonculturable bacteria ´. ACS Infect Dis.

[42] Zhang Y, Kepiro I, Ryadnov MG, Pagliara S (2023). Single cell killing kinetics differentiate phenotypic bacterial responses to different antibacterial classes. Microbiol Spectr.

[43] Mathews CK. 2015. *Bacteriophage T4*, p. 1–11. In eLS. John Wiley & Sons, Ltd, Chichester, UK.

[44] Koskella B, Brockhurst MA (2014). Bacteria-phage coevolution as a driver of ecological and evolutionary processes in microbial communities. FEMS Microbiol Rev.

[45] Endersen L, O’Mahony J, Hill C, Ross RP, McAuliffe O, Coffey A (2014). Phage therapy in the food industry. Annu Rev Food Sci Technol.

[46] Denes T, Wiedmann M (2014). Environmental responses and phage susceptibility in foodborne pathogens: implications for improving applications in food safety. Curr Opin Biotechnol.

[47] Strathdee SA, Hatfull GF, Mutalik VK, Schooley RT (2023). Phage therapy: from biological mechanisms to future directions. Cell.

[48] Abedon ST (2019). Phage-antibiotic combination treatments: antagonistic impacts of antibiotics on the pharmacodynamics of phage therapy?. Antibiotics.

[49] Conners R, McLaren M, Łapińska U, Sanders K, Stone MRL, Blaskovich MAT (2021). CryoEM structure of the outer membrane secretin channel pIV from the f1 filamentous bacteriophage. Nat Commun.

[50] Łapińska U, Glover G, Kahveci Z, Irwin NAT, Milner DS, Tourte M, Albers SV, Santoro AE, Richards TA, Pagliara S. Systematic comparison of unilamellar vesicles reveals that archaeal core lipid membranes are more permeable than bacterial membranes. PLoS Biol. 2023;21:e3002048. 10.1371/journal.pbio.3002048 PMC10072491 37014915

[51] Pagliara S, Chimerel C, Langford R, Aarts DGAL, Keyser UF (2011). Parallel sub-micrometre channels with different dimensions for laser scattering detection. Lab Chip.

[52] Lapinska U, Glover G, Capilla-lasheras P, Young AJ, Pagliara S (2019). Bacterial ageing in the absence of external stressors. Philos Trans R Soc B Biol Sci.

[53] Dettmer SL, Keyser UF, Pagliara S (2014). Local characterization of hindered Brownian motion by using digital video microscopy and 3D particle tracking. Rev Sci Instrum.

[54] Smith A, Metz J, Pagliara S (2019). MMHelper: an automated framework for the analysis of microscopy images acquired with the mother machine. Sci Rep.

[55] Bamford RA, Smith A, Metz J, Glover G, Titball RW, Pagliara S (2017). Investigating the physiology of viable but non-culturable bacteria by microfluidics and time-lapse microscopy. BMC Biol.

[56] Cama J, Al Nahas K, Fletcher M, Hammond K, Ryadnov MG, Keyser UF (2022). An ultrasensitive microfluidic approach reveals correlations between the physico-chemical and biological activity of experimental peptide antibiotics. Sci Rep.

[57] Stone MRL, Łapińska U, Pagliara S, Masi M, Blanchfield JT, Cooper MA (2020). Fluorescent macrolide probes – synthesis and use in evaluation of bacterial resistance. RSC Chem Biol.

[58] Blaskovich MA, Phetsang W, Stone MR, Lapinska U, Pagliara S, Bhalla R, et al. Antibiotic-derived molecular probes for bacterial imaging, p. 2. In Photonic Diagnosis and Treatment of Infections and Inflammatory Diseases II. 2019.

[59] Dimitriu T, Kurilovich E, Łapińska U, Severinov K, Pagliara S, Szczelkun MD (2022). Bacteriostatic antibiotics promote CRISPR-Cas adaptive immunity by enabling increased spacer acquisition. Cell Host Microbe.

[60] Wang P, Robert L, Pelletier J, Dang WL, Taddei F, Wright A (2010). Robust growth of Escherichia coli. Curr Biol.

[61] Athukoralage JS, White MF (2022). Cyclic nucleotide signaling in phage defense and counter-defense. Annu Rev Virol.

[62] Athukoralage JS, Graham S, Rouillon C, Grüschow S, Czekster CM, White MF (2020). The dynamic interplay of host and viral enzymes in type iii crispr-mediated cyclic nucleotide signalling. Elife.

[63] Cohen D, Melamed S, Millman A, Shulman G, Oppenheimer-Shaanan Y, Kacen A (2019). Cyclic GMP–AMP signalling protects bacteria against viral infection. Nature.

[64] Tal N, Morehouse BR, Millman A, Stokar-Avihail A, Avraham C, Fedorenko T (2021). Cyclic CMP and cyclic UMP mediate bacterial immunity against phages. Cell.

[65] Tal N, Millman A, Stokar-Avihail A, Fedorenko T, Leavitt A, Melamed S (2022). Bacteria deplete deoxynucleotides to defend against bacteriophage infection. Nat Microbiol.

[66] Maguin P, Varble A, Modell JW, Marraffini LA (2022). Cleavage of viral DNA by restriction endonucleases stimulates the type II CRISPR-Cas immune response. Mol Cell.

[67] Rostøl JT, Xie W, Kuryavyi V, Maguin P, Kao K, Froom R (2021). The Card1 nuclease provides defence during type III CRISPR immunity. Nature.

[68] Golec P, Wiczk A, Majchrzyk A, Łoś JM, Węgrzyn G, Łoś M (2010). A role for accessory genes rI.-1 and rI.1 in the regulation of lysis inhibition by bacteriophage T4. Virus Genes.

[69] Erez Z, Steinberger-Levy I, Shamir M, Doron S, Stokar-Avihail A, Peleg Y (2017). Communication between viruses guides lysis-lysogeny decisions. Nature.

[70] Stokar-avihail A, Tal N, Erez Z, Lopatina A (2019). Widespread utilization of peptide communication in phages infecting soil and pathogenic bacteria article widespread utilization of peptide communication in phages infecting soil and pathogenic bacteria. Cell Host Microbe.

[71] Tan D, Hansen MF, de Carvalho LN, Røder HL, Burmølle M, Middelboe M (2020). High cell densities favor lysogeny: induction of an H20 prophage is repressed by quorum sensing and enhances biofilm formation in Vibrio anguillarum. ISME J.

[72] Silpe JE, Bassler BL (2019). A host-produced quorum-sensing autoinducer controls a phage lysis-lysogeny decision. Cell.

[73] Laganenka L, Sander T, Lagonenko A, Chen Y, Link H, Sourjik V (2019). Quorum sensing and metabolic state of the host control lysogeny-lysis switch of bacteriophage T1. MBio.

[74] Berngruber TW, Froissart R, Choisy M, Gandon S (2013). Evolution of virulence in emerging epidemics. PLoS Pathog.

[75] Bruce JB, Lion S, Buckling A, Westra ER, Gandon S (2021). Regulation of prophage induction and lysogenization by phage communication systems. Curr Biol.

[76] Rollie C, Chevallereau A, Watson BNJ, Chyou TY, Fradet O, McLeod I (2020). Targeting of temperate phages drives loss of type I CRISPR–Cas systems. Nature.

[77] Wendling CC, Lange J, Liesegang H, Sieber M, Pöhlein A, Bunk B (2022). Higher phage virulence accelerates the evolution of host resistance. Proc R Soc B Biol Sci.

[78] Chao L, Chen CK, Shi C, Rang CU. Spatial and temporal distribution of ribosomes in single cells reveals aging differences between old and new daughters of Escherichia coli. bioRxiv Prepr. 2023. 10.1101/2023.06.21.545935. 10.7554/eLife.89543 PMC11578582 39565213

[79] Smith A, Kaczmar A, Bamford RA, Smith C, Frustaci S, Kovacs-Simon A (2018). The culture environment influences both gene regulation and phenotypic heterogeneity in Escherichia coli. Front Microbiol.

[80] Simmons EL, Bond MC, Koskella B, Drescher K, Bucci V, Nadell CD (2020). Biofilm structure promotes coexistence of phage-resistant and phage-susceptible bacteria. mSystems.

[81] Testa S, Berger S, Piccardi P, Oechslin F, Resch G, Mitri S (2019). Spatial structure affects phage efficacy in infecting dual-strain biofilms of Pseudomonas aeruginosa. Commun Biol.

