# Supplementary figures and images for: Slow growing bacteria survive bacteriophage in isolation
Source: ISME Commun. 2023 Sep 8;3:95. doi: 10.1038/s43705-023-00299-5 (PMC10491631; doi:10.1038/s43705-023-00299-5)

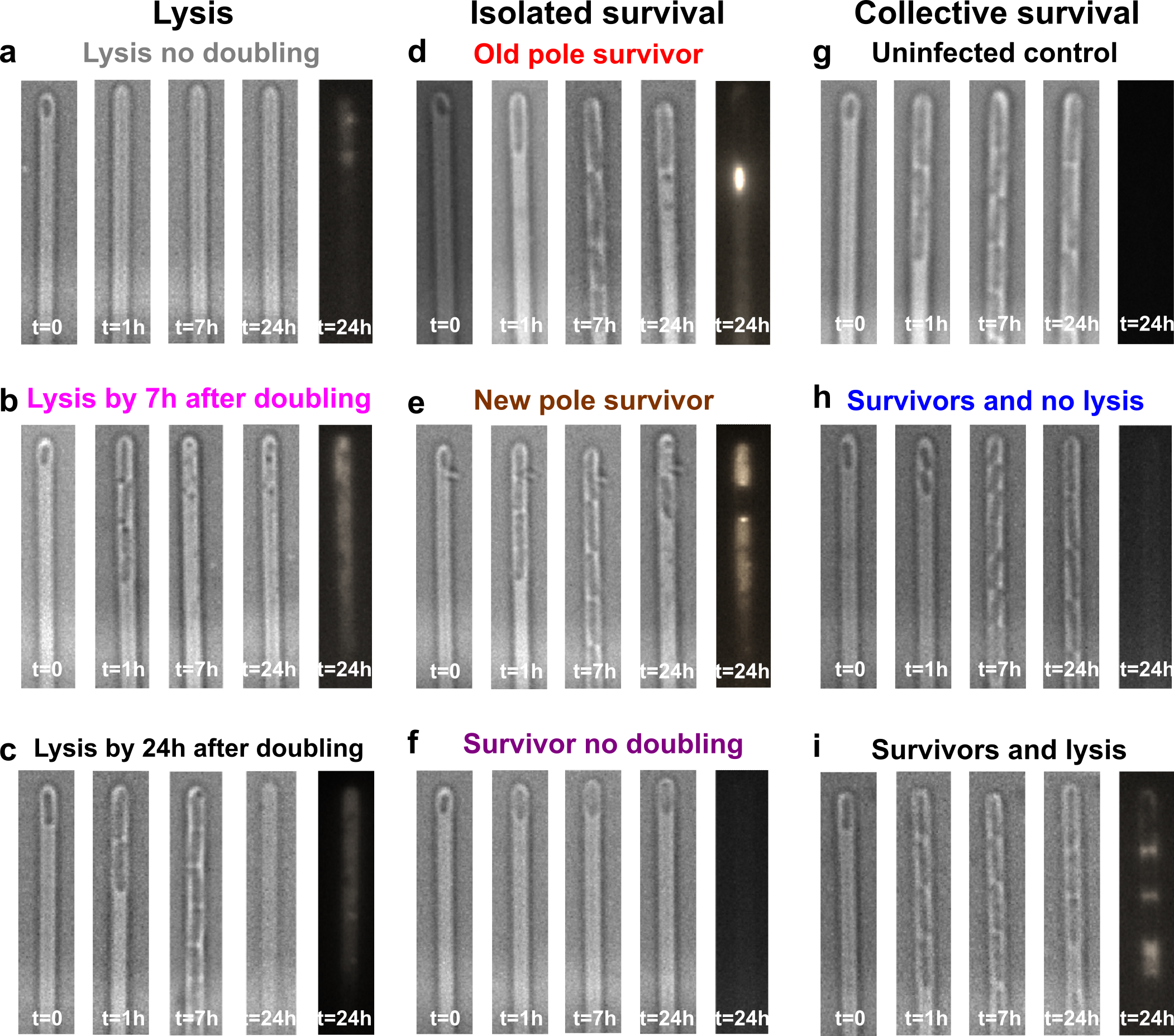

Supplement: Supplementary file 1 — Figure S1 [file 43705_2023_299_MOESM1_ESM.tif]

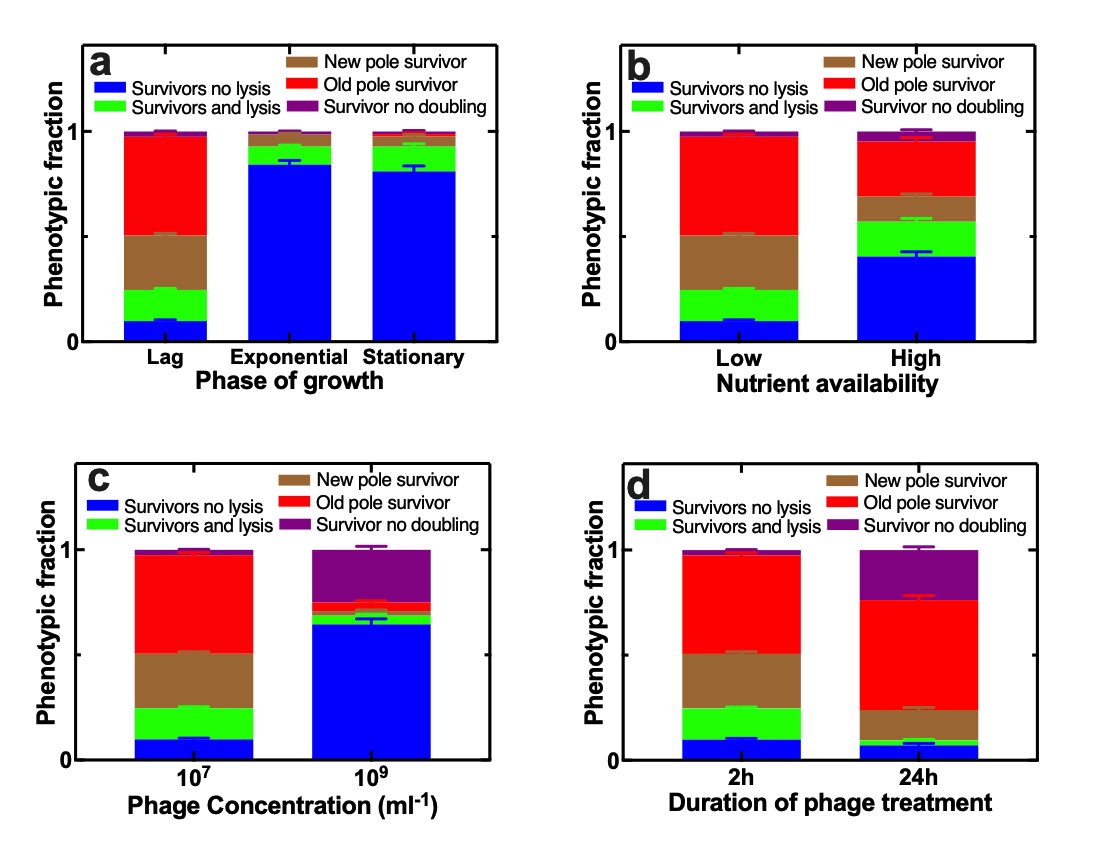

Supplement: Supplementary file 2 — Figure S2 [file 43705_2023_299_MOESM2_ESM.tif]

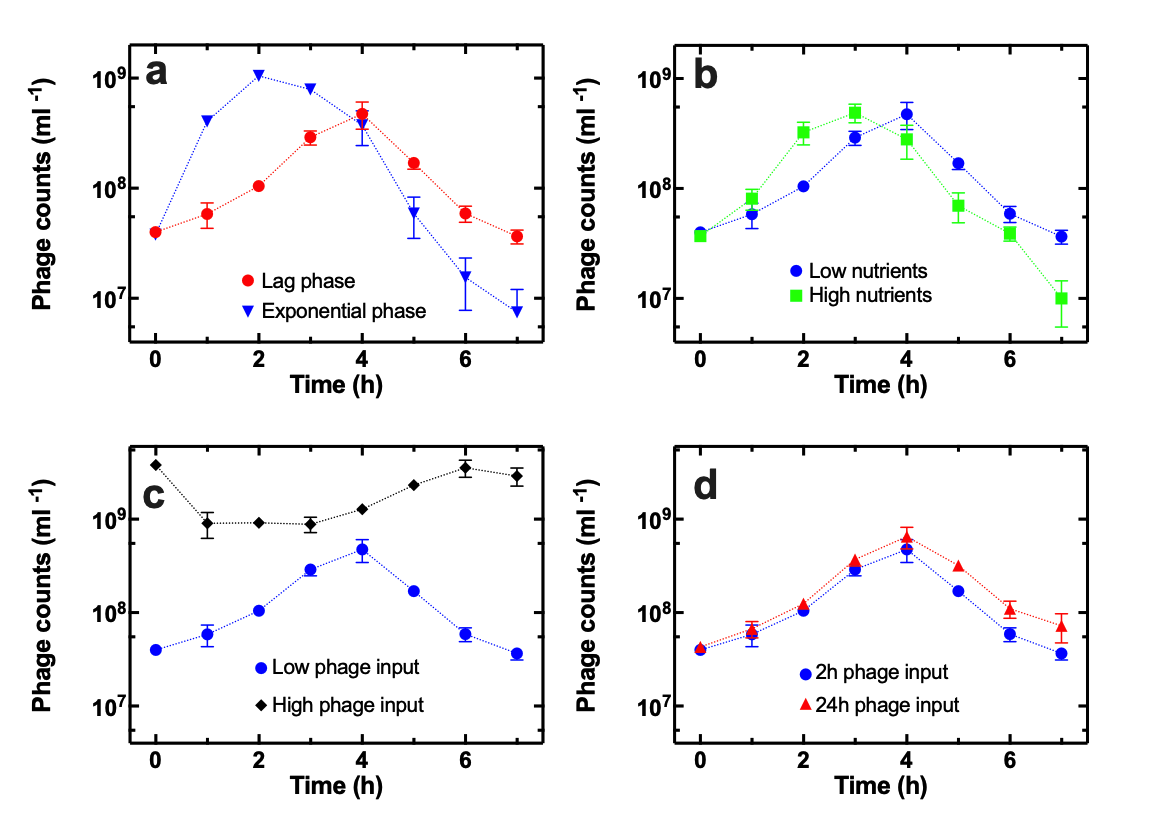

Supplement: Supplementary file 3 — Figure S3 [file 43705_2023_299_MOESM3_ESM.tif]

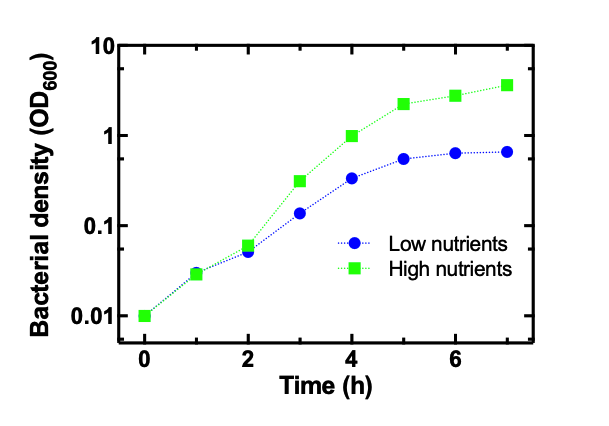

Supplement: Supplementary file 4 — Figure S4 [file 43705_2023_299_MOESM4_ESM.tif]

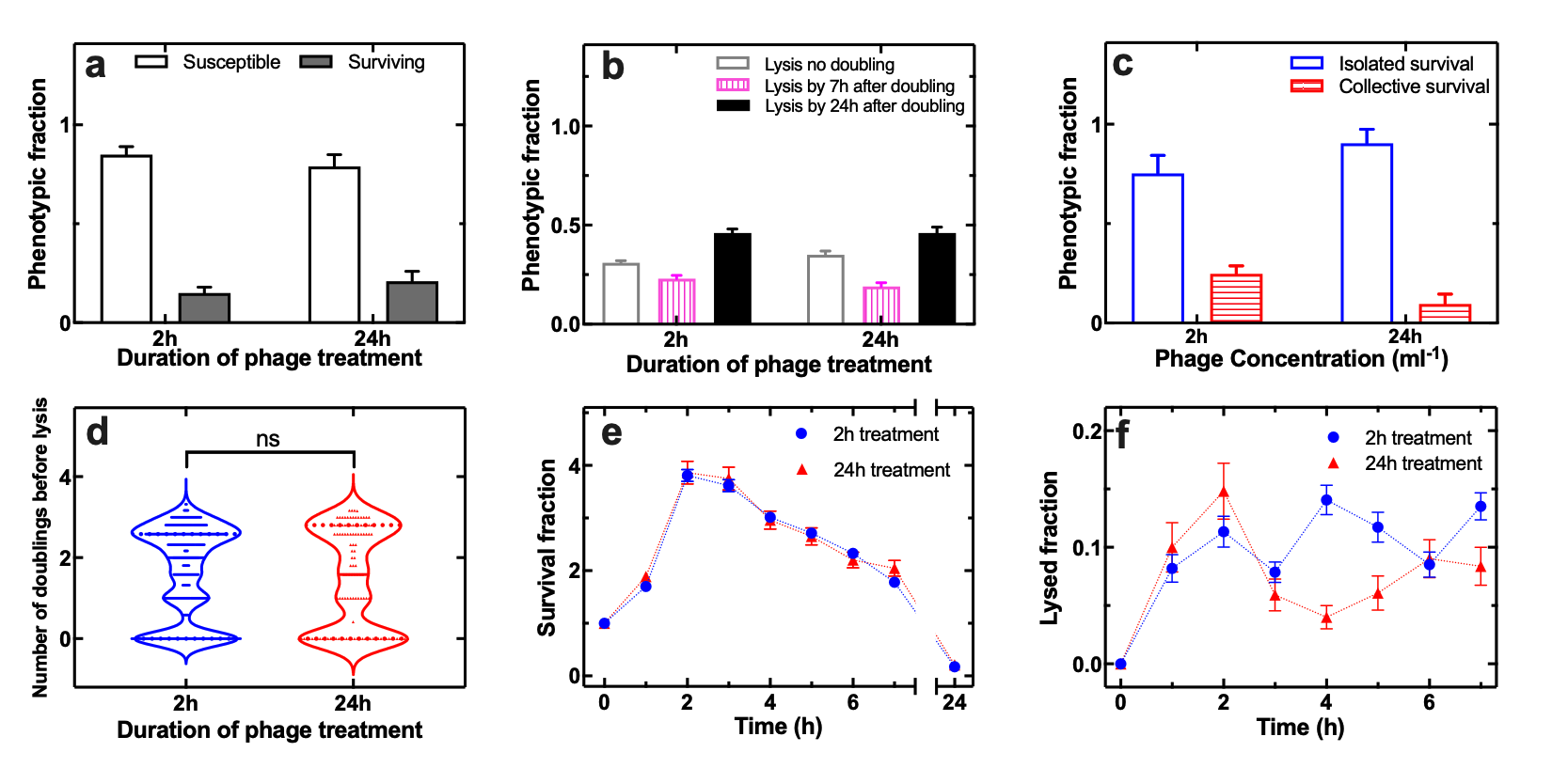

Supplement: Supplementary file 5 — Figure S5 [file 43705_2023_299_MOESM5_ESM.tif]

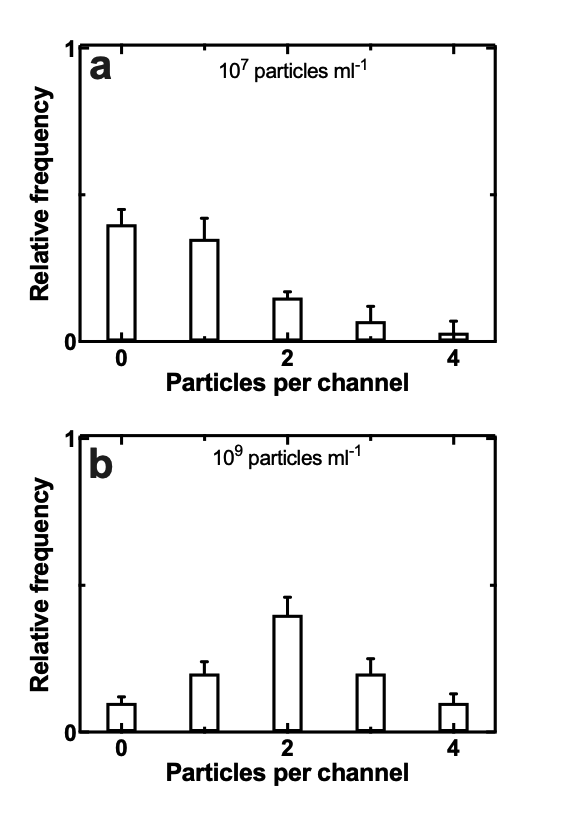

Supplement: Supplementary file 6 — Figure S6 [file 43705_2023_299_MOESM6_ESM.tif]

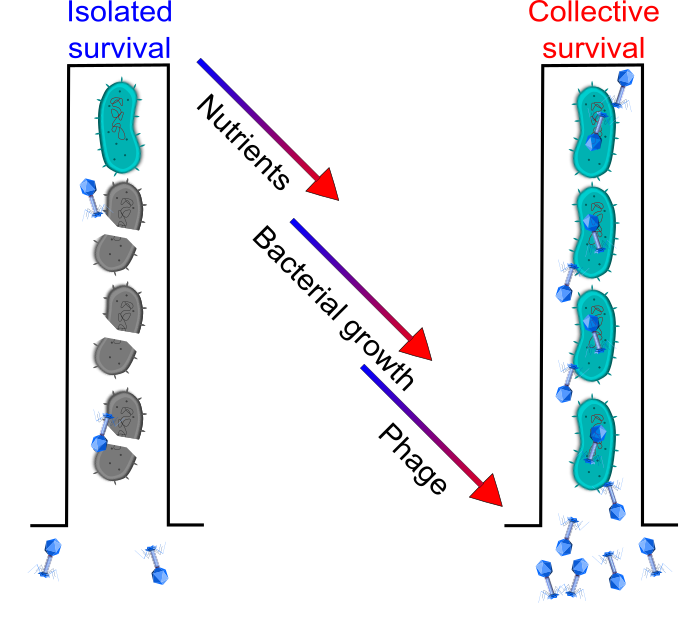

Supplement: Supplementary file 7 — Figure S7 [file 43705_2023_299_MOESM7_ESM.tif]
